# The right heart perspective in chronic cardiorenal syndrome: the key role of right heart function and tricuspid regurgitation innovation

**DOI:** 10.3389/fcvm.2025.1710898

**Published:** 2026-01-12

**Authors:** Xueshi Yin, Long Tang, Jianping Liu, Yongheng Zhang

**Affiliations:** 1 Department of Cardiovascular Surgery, Suining Central Hospital, Suining, Sichuan, China; 2 Department of Clinical Medicine, North Sichuan Medical College, Nanchong, Sichuan, China

**Keywords:** cardiorenal syndrome (CRS), renal venous congestion, right ventricular dysfunction (RV dysfunction), transcatheter tricuspid intervention, tricuspid regurgitation (TR)

## Abstract

Cardiorenal syndrome (CRS) refers to the pathophysiological interaction between cardiac dysfunction and kidney injury. Traditional CRS research has focused primarily on the impact of left heart failure on renal function. However, increasing evidence suggests that abnormalities in right heart function, particularly tricuspid regurgitation (TR), critically exacerbate the progression of CRS by promoting renal venous congestion, worsening kidney function, and further aggravating right heart failure. With the aging population and prolonged survival of patients with heart failure, the prevalence of TR has significantly increased and has a substantial impact on prognosis. Therefore, there is an urgent need to reassess the role of TR in heart–kidney interactions. This review summarizes the pathophysiology, clinical evidence, and treatment strategies of TR in the context of CRS, with the aim of raising awareness of the right-heart-centered perspective. Kidney injury caused by right heart dysfunction is driven by multiple mechanisms, among which elevated right atrial pressure and consequent renal venous congestion appear to be more important than reduced renal perfusion caused by low cardiac output alone. In patients with moderate or severe TR, renal function deteriorates significantly, whereas interventional treatment that reduces TR can improve right heart function and lower the risk of adverse events. Future research should challenge the traditional left-heart-dominant paradigm, focusing on mechanistic studies, early assessment and risk stratification, interventional therapy, and the synergistic effects of new drug combinations. Addressing current limitations and research gaps is crucial to overcoming therapeutic bottlenecks and improving long-term outcomes in patients with chronic cardiorenal syndrome.

## 1 Introduction

**Figure 1:**
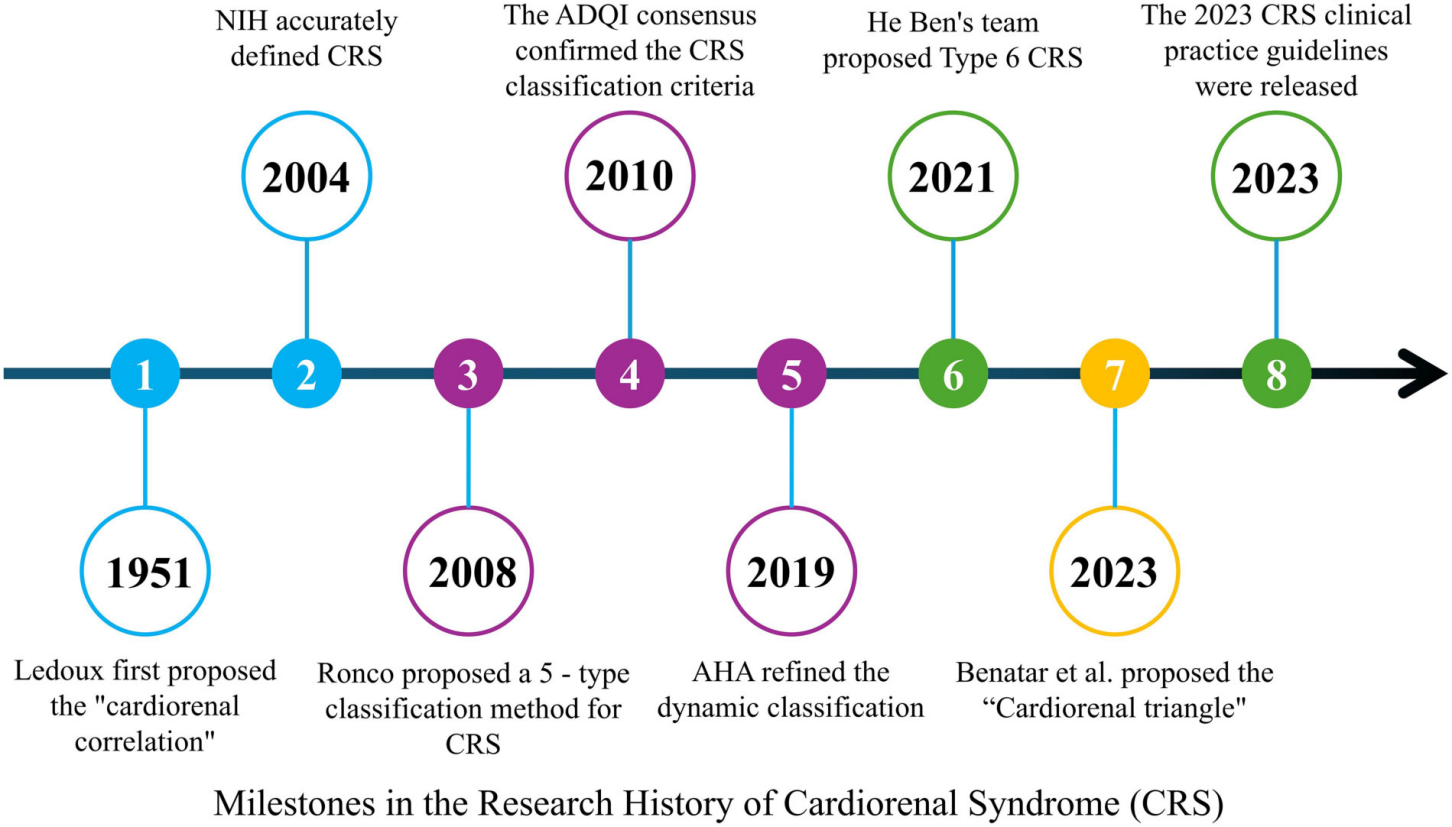
Milestones in the research history of cardiorenal syndrome (CRS). A timeline summarizing key milestones in CRS research, from Ledoux's initial proposal of the “cardiorenal correlation” in 1951, to the NIH definition of CRS in 2004, the five-type classification by Ronco et al. in 2008, the dynamic refinement by the AHA in 2019, the proposal of CRS type 6 by He Ben in 2021, and the publication of the 2023 clinical practice guidelines for CRS.

The concept of heart–kidney interaction can be traced back to clinical observations by Ledoux in 1951, which described the mutual influence between cardiac and renal dysfunction (1). In 2004, the National Institutes of Health (NIH) formally defined this bidirectional interaction as cardiorenal syndrome (CRS) (2). Although He Ben and colleagues later proposed a chronic secondary form of CRS (CRS type 6) (3), the five-type classification system proposed by Ronco et al. in 2008 remains widely used in clinical practice. This system categorizes CRS into five types based on pathogenesis and temporal characteristics, with the most common forms being kidney dysfunction caused by acute or chronic heart failure (CRS types 1 and 2) (Figure 1) (4–6).

Traditional CRS research has primarily focused on left ventricular (LV) dysfunction and reduced cardiac output. However, existing evidence suggests that left ventricular ejection fraction (LVEF) does not clearly correlate with estimated glomerular filtration rate (eGFR) (7). In contrast, right heart dysfunction has long been under-recognized in the CRS paradigm. Tricuspid regurgitation, often regarded as a secondary manifestation of right heart failure, has frequently been considered to have limited independent clinical significance.

Benatar and colleagues recently introduced the concept of a “TR–right ventricular function–renal congestion” cardiorenal triangle, describing how TR leads to right ventricular (RV) dysfunction and venous congestion, which in turn results in renal dysfunction (8). Emerging data suggest that TR is not only a hallmark of right heart failure but may also act as a major driving factor in heart–kidney dysfunction. The strong association between TR and adverse outcomes in CRS has become increasingly evident as the prevalence of TR rises with population aging and improved survival in heart failure (9, 10). This poses a significant threat to public health.

Therefore, it is imperative to reassess the role of TR in heart–kidney interactions and to recognize the critical contribution of right heart function—particularly TR—to chronic cardiorenal syndrome (Table 1).

**Table 1:** Based on ronco et al.'s five-type CRS classification.

Type	Name	Pathophysiology	Typical clinical manifestations	Common triggers/underlying causes	Treatment strategies
CRS-1	Acute Cardiorenal Syndrome	Acute heart failure leading to renal hypoperfusion, causing AKI	Oliguria, increased blood creatinine 24–72 h post-acute heart failure or myocardial infarction	Acute myocardial infarction, decompensated heart failure	Optimize cardiac output; Diuretics; RRT if necessary
CRS-2	Chronic Cardiorenal Syndrome	Chronic heart failure leading to long-term low perfusion, accelerating CKD progression	Progressive eGFR decline ≥3 months, proteinuria	Dilated cardiomyopathy, etc.	RAAS inhibitors, Beta-blockers, Volume management
CRS-3	Acute Renal-Heart Syndrome	AKI causing volume overload, leading to acute myocardial injury/arrhythmia	Acute pulmonary edema, ventricular arrhythmias after AKI	Ischemic AKI, etc.	Remove renal injury triggers; Volume control; Dialysis support
CRS-4	Chronic Renal-Heart Syndrome	CKD causing uremic toxins that accelerate cardiovascular remodeling	CKD patients with left ventricular hypertrophy	Diabetic nephropathy, etc.	Antihypertensive treatment, Anemia correction, Calcium-phosphate metabolism regulation, Cardiovascular protection
CRS-5	Acute Secondary Cardiorenal Syndrome	Systemic diseases triggering inflammatory storms leading to concurrent heart and kidney dysfunction	Multiple organ failure	Sepsis, SLE, etc.	Control primary disease; Organ support

## 2 Pathophysiological mechanisms of heart-kidney interaction from the right heart perspective

From the right heart perspective, kidney injury in chronic heart failure is driven by multiple interrelated mechanisms, with hemodynamic remodeling as the initiating event. In contrast to the traditional left-heart-centered view, elevated right atrial pressure and renal venous congestion (“backward failure”) appear to be more crucial than reduced renal perfusion pressure due to low cardiac output (“forward failure”).

Clinical studies have demonstrated that LVEF does not have a direct correlation with eGFR (7). Data from the ESCAPE trial confirmed that increased renal interstitial hydrostatic pressure can compress peritubular capillaries, directly damaging renal parenchyma and impairing filtration (11). Persistent elevation of central venous pressure (CVP) thus contributes significantly to renal dysfunction.

Right ventricular volume overload leads to leftward displacement of the interventricular septum, limiting LV diastolic filling and further lowering cardiac output. This creates a vicious cycle of “low cardiac output–high venous pressure,” exacerbating systemic tissue hypoperfusion and pre-renal injury. Clinically, this is manifested as diuretic resistance and abnormal redistribution of cortical–medullary blood flow (7).

At the neurohormonal and inflammatory level, renal congestion triggers activation of the renin–angiotensin–aldosterone system (RAAS). Angiotensin II maintains compensatory GFR by constricting efferent arterioles but at the cost of glomerular hyperfiltration damage. Along with aldosterone, angiotensin II promotes cardiac and renal fibrosis through TGF-*β*–mediated pathways. Venous congestion also induces endothelial dysfunction (eNOS uncoupling with reduced NO and increased ROS) and systemic inflammatory responses (12), accelerating cardiomyocyte and renal tubular cell apoptosis as well as epithelial–mesenchymal transition (EMT) via the NF-*κ*B pathway.

**Figure 2:**
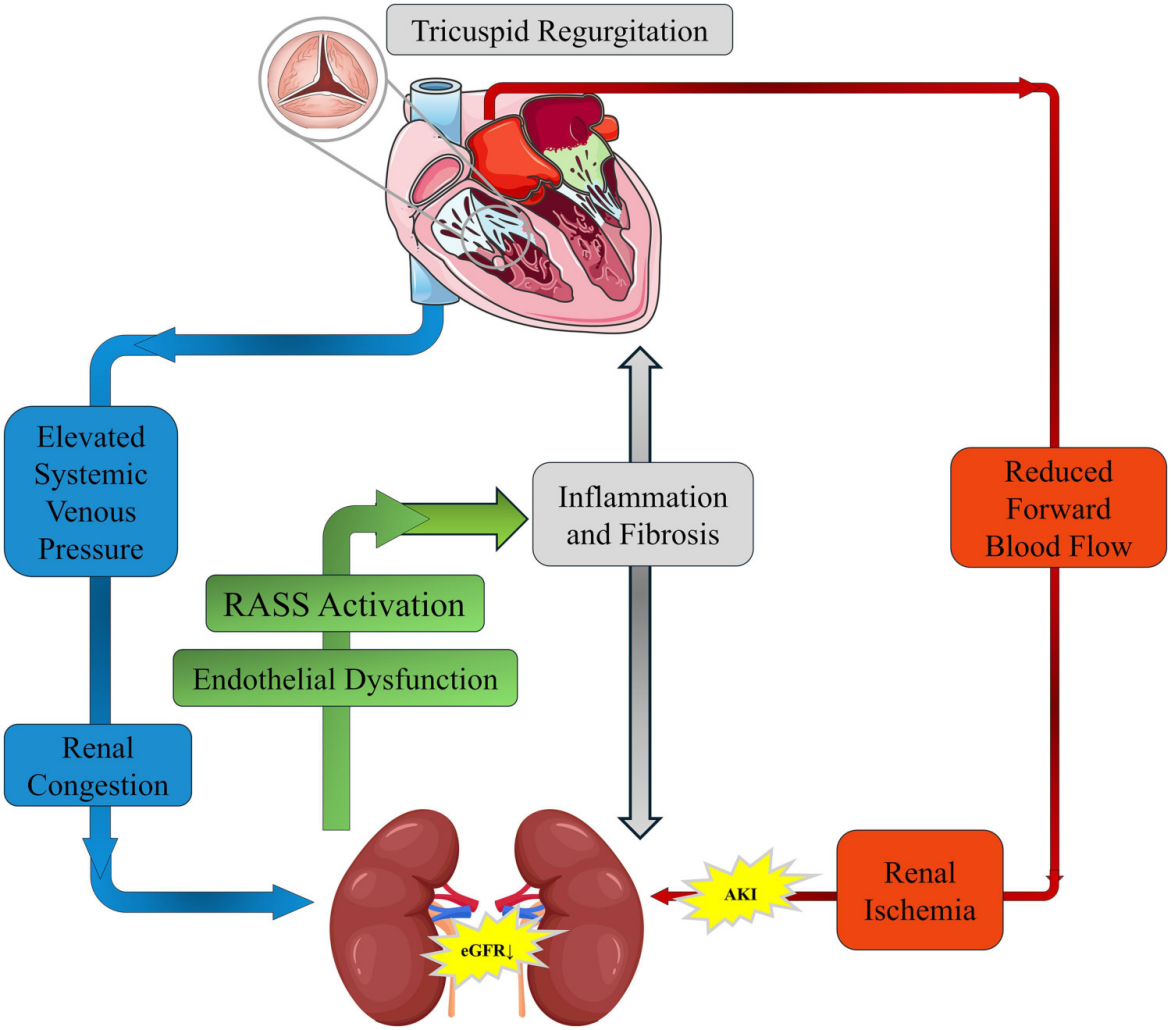
Pathophysiological mechanisms of tricuspid regurgitation (TR) in cardiorenal syndrome (CRS). Schematic representation of how TR exacerbates CRS by increasing systemic venous pressure and causing renal congestion, leading to RAAS activation, inflammation, and fibrosis. Concurrently, reduced forward blood flow results in renal ischemia and AKI. These mechanisms interact to create a vicious cycle that progressively impairs both cardiac and renal function.

Anemia and dysregulation of the erythropoietin (EPO) axis further contribute to metabolic decompensation. Right-heart-associated hepatic congestion upregulates hepcidin, leading to functional iron deficiency (13). Chronic inflammation blunts EPO receptor sensitivity, diminishing the dual protective effects of the PI3 K/Akt pathway in suppressing apoptosis and fibrosis in both cardiac and renal tissues (14, 15). Altogether, these processes form a “congestion–fibrosis–metabolic imbalance” closed-loop progression model (Figure 2).

## 3 Clinical evidence of TR-related cardiorenal syndrome

### 3.1 Relationship between tricuspid regurgitation and right heart function

Tricuspid regurgitation is both an important clinical manifestation and a marker of right heart dysfunction. Most TR is functional or secondary, with pathophysiological mechanisms primarily linked to RV dysfunction and right atrial (RA) dilation. Right ventricular dysfunction leads to RV dilation and geometric distortion, stretching the tricuspid annulus and impairing coaptation of the valve leaflets. Right atrial enlargement further exacerbates TR severity, perpetuating a vicious cycle (16).

TR, in turn, worsens right heart dysfunction. The more severe the TR, the higher the risk of RV failure and poor prognosis. In an echocardiographic study by Towheed et al. (17), TR significantly impacted postoperative outcomes following left-sided valvular surgery by worsening RV dysfunction. The incidence of RV dysfunction in patients with TR was noticeably higher than in those without TR (28.7% vs. 18.4%, *P* < 0.05), and the postoperative incidence of heart failure was also higher (15.6% vs. 6.2%, *P* < 0.05). Moreover, the 30-day mortality rate in patients with moderate-to-severe TR was significantly higher compared to patients with mild or no TR (25.3% vs. 10.2% and 3.8%, respectively; *P* < 0.05).

Conversely, reducing TR can improve right heart function and clinical outcomes. Tanaka et al. (18) reported that in patients with RV dysfunction (RVFAC <35%) undergoing transcatheter tricuspid edge-to-edge repair (T-TEER), those with improved RV function (increase in RVFAC) had a significantly lower risk of the composite endpoint of death or heart failure hospitalization within one year (HR 0.35; 95% CI 0.14–0.89; *P* = 0.028). Patients with smaller baseline RV diameter and greater reduction in TR were more likely to experience improvement in RV function, underscoring the prognostic relevance of effective TR reduction.

### 3.2 Relationship between tricuspid regurgitation and renal function

TR can also accelerate kidney injury through two main mechanisms: elevated systemic venous pressure and reduced effective forward flow. Elevated systemic venous pressure results from increased right atrial pressure and CVP, leading to retrograde transmission of pressure to the renal veins. This renal venous congestion reduces the net filtration pressure across the glomerular capillaries, thereby decreasing GFR and causing kidney dysfunction. In parallel, reduced cardiac output due to RV dysfunction further compromises renal perfusion.

Historically, insufficient renal perfusion due to elevated CVP has been referred to as “renal congestion,” while renal hypoperfusion related to left heart pump failure is classically termed “cardiorenal syndrome.” However, these mechanisms often coexist and interact in patients with combined right and left heart disease.

A cohort study by Butcher et al. (19) showed that 45% of patients with moderate or severe TR had significant kidney dysfunction (eGFR <60 mL/min/1.73 m^2^). The presence of RV dysfunction (TAPSE <14 mm) further increased the risk of renal injury (OR 1.49; 95% CI 1.11–1.99; *P* = 0.008), indicating that TR is independently associated with kidney impairment and that RV–renal coupling is clinically important.

Further analysis demonstrated that renal function parameters in patients with moderate-to-severe TR were significantly worse than in those with mild TR (eGFR 37.2 vs. 45.6 mL/min/1.73 m^2^, Δ=8.4; *P* < 0.001; serum urea nitrogen 21.4 vs. 16.8 mg/dL; *P* < 0.001). These patients also showed RA enlargement (RA volume index >40 mL/m^2^) and elevated right ventricular systolic pressure (RVSP ≥40 mmHg), reflecting the integrated impact of right-sided hemodynamics on renal function (19).

Chen et al. (20) reported that even when cardiac output was not significantly reduced, elevated CVP independently contributed to the development of acute kidney injury (AKI). For every 1 mmHg increase in CVP, the risk of AKI increased by 9.2% (OR 1.092; 95% CI 1.047–1.138), providing direct evidence for the role of CVP-mediated renal congestion.

In a large retrospective cohort of 11,135 critically ill patients, Sun et al. (21) found that those with CVP >13.2 mmHg (highest quartile) had a 2.1-fold higher risk of AKI compared with those with CVP ≤8.29 mmHg (OR 3.12; 95% CI 2.78–3.50). When CVP ≥15 mmHg, the rate of renal function deterioration accelerated markedly (*P* < 0.001), suggesting a pathological threshold effect for elevated CVP. Collectively, these studies highlight that the backward pressure effect of TR on the kidney may be more deleterious than forward flow reduction alone.

## 4 Treatment strategies and approaches from the right heart perspective

Management of CRS has traditionally emphasized pharmacological therapies aimed at reducing cardiac load, improving LV function, and preserving renal function. From a right-heart-centered perspective, however, both the limitations of conventional therapies and the emerging role of TR-targeted interventions must be reconsidered.

### 4.1 Pharmacological therapies

The mainstay treatments in chronic heart failure include angiotensin-converting enzyme inhibitors (ACEIs), angiotensin receptor blockers (ARBs), angiotensin receptor–neprilysin inhibitors (ARNIs), β-blockers, and mineralocorticoid receptor antagonists. Although these agents improve LV remodeling and overall prognosis, their effect on TR and right-sided congestion is often indirect and limited.

Loop diuretics (e.g., furosemide) rapidly relieve systemic congestion by reducing preload, but excessive or chronic use may lead to intravascular volume depletion and prerenal hypoperfusion. This, in turn, can worsen kidney function through RAAS activation and sympathetic overdrive (22). Clinical data indicate that for every 40 mg/day increase in loop diuretic dosage, the annual rate of eGFR decline increases by approximately 1.2 mL/min/1.73 m^2^ (22). Long-term high-dose diuretic use may also induce electrolyte disturbances (e.g., hypokalemia, hypomagnesemia) and vacuolar degeneration of renal tubular epithelial cells, promoting tubulointerstitial fibrosis (23).

RAAS inhibitors (ACEIs/ARBs, ARNIs) reduce cardiovascular events and mitigate LV remodeling; however, their dose-dependent hypotension and reduction in renal perfusion pressure may accelerate renal function decline in susceptible patients (24). The MERIT-HF trial (25) systematically excluded patients with eGFR <30 mL/min/1.73 m^2^, resulting in limited evidence regarding the safety and efficacy of *β*-blockers and other standard heart failure therapies in those with moderate-to-severe chronic kidney disease (CKD). In real-world practice, approximately one-third of patients with CRS type 2 must reduce or discontinue disease-modifying drugs because of hypotension, hyperkalemia, or worsening renal function, creating a “therapeutic ceiling” (26).

Newer agents such as sodium–glucose cotransporter 2 inhibitors (SGLT2i) and soluble guanylate cyclase (sGC) stimulators have been shown to delay renal function decline and improve outcomes in chronic heart failure (27). However, their direct impact on TR severity and right-sided venous congestion remains to be clarified, and TR-specific pharmacotherapy is still lacking.

### 4.2 Interventional and surgical therapies

The indications for surgical tricuspid valve repair or replacement have traditionally been narrow, and perioperative risk is high, particularly in patients with advanced RV dysfunction, severe pulmonary hypertension, or multiple comorbidities. As a result, many patients with significant TR are referred late or not considered for surgery.

With the advent of transcatheter techniques, interventional treatment for TR has become an important therapeutic option. Transcatheter tricuspid valve repair or replacement, including edge-to-edge repair systems such as TriClip, has shown promising results in reducing TR severity and improving functional status in high-risk patients (28). Early data suggest that effective reduction in TR may translate into improved right heart function, relief of venous congestion, and potentially better renal outcomes, although long-term data in CRS populations are still limited.

Surgical repair or replacement remains an effective option for selected patients with severe TR, particularly when performed early, before irreversible RV remodeling and organ dysfunction occur. Early surgery can reverse right heart dysfunction and improve survival, especially in patients undergoing concomitant left-sided valve surgery (29, 30). The challenge lies in refining patient selection, timing, and perioperative management to optimize outcomes.

## 5 Future perspectives

### 5.1 Early assessment of right heart function

Early assessment of right heart function requires integration of multimodality imaging and circulating biomarkers. In echocardiography, tricuspid annular plane systolic excursion (TAPSE; <1.6 cm indicating poor prognosis) and the right ventricular–pulmonary artery coupling ratio [TAPSE/systolic pulmonary artery pressure (SPAP)] are key parameters for monitoring RV systolic function (31–33). Three-dimensional echocardiography can more accurately characterize tricuspid valve morphology and mechanisms of functional TR, offering advantages over two-dimensional imaging for preprocedural planning (34).

Cardiac magnetic resonance (CMR), as the gold standard for right heart volumetric assessment, can quantify RV size, function, and remodeling, and is particularly useful in patients with severe TR or complex congenital anatomy (35–37).

In terms of biomarkers, CA-125 can independently predict venous congestion (38), while bioactive adrenomedullin (bioADM) reflects congestion and biventricular filling pressures (39). Soluble ST2 (sST2) is associated with myocardial fibrosis and remodeling (40), and urinary albumin excretion may reflect the severity of renal congestion. Novel AKI biomarkers such as TIMP-2×IGFBP7, neutrophil gelatinase-associated lipocalin (NGAL), and cystatin C can further refine multi-organ function assessment and provide early warning signals of kidney injury in CRS (41, 42).

### 5.2 Multi-angle, multi-indicator clinical trial research

Clinical studies have shown that RV functional parameters and pulmonary hemodynamics are strong predictors of postoperative outcomes. Masiero et al. reported that for every 10 mmHg increase in pulmonary artery pressure or every 5 mm decrease in TAPSE, the risk of mortality after mitral valve transcatheter edge-to-edge repair increased by 17% and 18%, respectively (43). A meta-analysis including 8,672 patients found that pulmonary hypertension (HR = 1.70) and TR significantly increased mortality risk after transcatheter mitral valve repair; each 10 mmHg increase in systolic pulmonary artery pressure was associated with a 17% increase in relative risk (44).

These findings underscore the importance of dynamic preoperative monitoring of right heart function (e.g., TAPSE, RV–pulmonary artery coupling, pulmonary pressures) for risk stratification. Future clinical trials should incorporate multi-angle and multi-indicator assessment of right heart function, including TR severity, RV mechanics, venous congestion markers, and renal outcomes, to better define optimal therapeutic strategies.

### 5.3 Therapeutic innovation and personalized strategies

Pharmacological treatment currently focuses on diuretics to relieve congestion. Vasodilators such as phosphodiesterase-5 inhibitors may benefit patients with pulmonary hypertension and RV dysfunction (29). However, TR-specific pharmacologic agents remain an unmet need.

In the interventional field, transcatheter tricuspid edge-to-edge repair (TEER) significantly reduces TR severity and has been associated with lower mortality and fewer heart failure hospitalizations in high-risk populations (30, 45). Patients achieving TR ≤1 + after TEER tend to have better outcomes, suggesting that the degree of TR reduction is clinically meaningful. Ongoing research aims to refine device technology, procedural techniques, and patient selection criteria.

Surgical treatment (tricuspid valve repair or replacement) continues to play an important role, especially when performed earlier in the disease course. Future efforts should focus on integrating surgical and transcatheter strategies, optimizing the timing of referral, and combining structural interventions with guideline-directed medical therapy and CRS-specific care pathways.

### 5.4 Current limitations and research gaps

Despite increasing recognition of the right-heart-centered perspective in CRS, several important limitations and knowledge gaps remain:

#### 5.4.1 Lack of randomized controlled trials (RCTs)

Most evidence linking TR, RV dysfunction, and renal outcomes comes from observational studies, registries, or *post hoc* analyses. High-quality RCTs specifically evaluating TR-targeted therapies in CRS populations are scarce.

#### 5.4.2 Heterogeneous assessment of TR and RV function

Definitions of TR severity, imaging protocols, and cutoffs (e.g., for TAPSE or RVFAC) vary across studies, limiting comparability and generalizability. Standardized, guideline-based assessment is needed.

#### 5.4.3 Limited long-term data on transcatheter TR interventions

While early results of TEER and transcatheter replacement are promising, long-term effects on survival, renal function, and hospitalization rates in CRS patients are not yet well established.

#### 5.4.4 Incomplete mechanistic understanding

The precise pathways linking renal venous congestion to tubulointerstitial fibrosis, metabolic dysregulation, and progression from AKI to CKD in CRS remain incompletely understood, particularly in the presence of chronic systemic inflammation.

#### 5.4.5 Lack of right-heart-specific biomarkers

Although several biomarkers reflect congestion or fibrosis, few are specific to right-sided hemodynamics or RV–renal interaction. Development and validation of such biomarkers could significantly enhance risk stratification and treatment guidance.

#### 5.4.6 Optimal timing of TR intervention is unclear

It remains uncertain at which stage of RV remodeling, pulmonary hypertension, or renal impairment TR intervention provides maximal benefit with acceptable risk. Prospective studies focusing on timing thresholds are needed.

Addressing these gaps will be essential to move from descriptive associations toward precise, mechanism-based, and individualized therapeutic strategies in chronic cardiorenal syndrome.
